# Supplementary material for: Lifelines COVID-19 cohort: investigating COVID-19 infection and its health and societal impacts in a Dutch population-based cohort
Source: BMJ Open. 2021 Mar 17;11(3):e044474. doi: 10.1136/bmjopen-2020-044474 (PMC7977080; doi:10.1136/bmjopen-2020-044474)
Supplement: Supplementary data [file bmjopen-2020-044474supp001.pdf]

Supplementary Table 1. Populations and COVID-19 infections, hospitalizations and deaths for the Netherlands as whole and for the Northern Provinces. Population values are as of January 1, 2020, retrieved from the Central Bureau Statistiek (https://opendata.cbs.nl/#/CBS/nl/dataset/03759ned/table; accessed July 10, 2020). COVID-19 statistics are as of June 09, 2020. Source: RIVM, downloaded from the CoronaWatchNL Github.

**Region**	**Population**	**Infections**	**Hospitalizations**	**Deaths**
Netherlands	17,181,084	47,903	11,800	6,031
North NL	1,729,505	1,491	320	122
Groningen	585,866	352	74	17
Friesland	493,682	616	130	65
Drenthe	649,957	523	116	40
